# Programa Geração Biz, Mozambique: how did this adolescent health initiative grow from a pilot to a national programme, and what did it achieve?

**DOI:** 10.1186/1742-4755-12-12

**Published:** 2015-02-17

**Authors:** Venkatraman Chandra-Mouli, Susannah Gibbs, Rita Badiani, Fernandes Quinhas, Joar Svanemyr

**Affiliations:** Department of Reproductive Health and Research, World Health Organization, Geneva, Switzerland; Department of Population, Family and Reproductive Health, Johns Hopkins Bloomberg School of Public Health, Baltimore, MD USA; Pathfinder International, Maputo, Mozambique; Government of Mozambique Ministry of Health, Maputo, Mozambique

## Abstract

Adolescent sexual and reproductive health gained particular traction in Mozambique following the 1994 International Conference on Population and Development leading to the inception of Programa Geração Biz (PGB), a multi-sectoral initiative that was piloted starting in 1999 and fully scaled-up to all provinces by 2007. We conducted a systematic review of the literature to gather information on PGB and analyzed how it planned and managed the scale-up effort using the WHO-ExpandNet framework. PGB’s activities comprised a clear and credible innovation. Appropriate resource and user organizations further facilitated national scale-up. Challenges relating to the complex nature of the multi-sectoral approach and resistance due to norms about adolescent sexual and reproductive health hindered scaling-up in some geographic areas. The national government exhibited commitment and ownership to PGB through budgetary support and integration into multiple policies. This study adds to the documentation of successful scaling-up strategies that can provide guidance for policy makers and programme managers.

## Introduction

This paper describes the inception and nationwide scale up of Programa Geração Biz^a^ (PGB), a multicomponent initiative aimed at improving the sexual and reproductive health of adolescents in Mozambique; analyzes what helped and hindered the scale up effort; and sets out the results achieved at the programme, health behaviours, and health outcomes levels.

PGB has achieved what many other adolescent sexual and reproductive health (ASRH) programmes in Africa have not i.e. large scale and sustained scale up of a complementary set of interventions. While a number of descriptions of the PGB and its achievements have been published [1–5] and its scale up process has been briefly examined in the context of a larger five-country comparison [6], there is a need to further analyze its scale up and the context in which that scale up occurred. The objective of this analysis is to inform policy makers, programme managers and international organizations operating in Africa and elsewhere about a successful initiative and how it was realized, with the hope that it will inspire those who are working hard to move beyond small-scale and short-lived projects.

This paper has two objectives – firstly to examine what factors helped and hindered the scale up of PGB, and secondly to examine what was achieved as a result of this programme.

## Methods

We gathered reports about PGB from UN agencies and Pathfinder International that describe the conception, inception, phased implementation, and monitoring and evaluation of the initiative. In addition, we carried out a systematic review of the literature on ASRH in Mozambique for information on the context in which these activities took place. In order to identify peer-reviewed literature, we used six academic databases including Scopus, Popline, PubMed, CINAHL, Embase, and African Index Medicus using combinations of search terms including “Mozambique,” “adolescent,” “youth,” “young,” “reproductive,” “sexual,” and “sex.”

Our searches of the academic databases yielded 630 unique citations. We reviewed all 630 titles and abstracts for pertinence to ASRH in Mozambique and selected 39 for full text review according to the following criteria:

-  Original research conducted in Mozambique or secondary data analysis published in a peer-reviewed journal;
-  Examines sexual and reproductive health knowledge, attitudes, behaviour, or outcomes or describes a sexual and reproductive health intervention
-  Adolescents are the primary study population or results are reported separately for adolescents
-  Full text available in English

Of the 39 full text articles, reviewed 22 were relevant and are included in this paper. These papers describe the state of ASRH in Mozambique prior to the inception of PGB (n = 5), during PGB scale-up (n = 6) and after scale-up (n = 3). In addition, several papers report on other ASRH interventions implemented in Mozambique (n = 5) or on PGB itself (n = 3).

We were able to get numerous reports and programme evaluations and have referenced them throughout this paper. These include narrative descriptions of PGB’s design and implementation process [1, 3, 4] as well as reports from evaluations that were conducted over the years [7–9].

**Figure 1:**
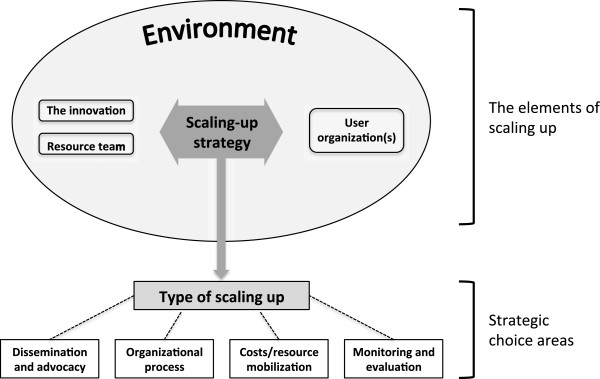
**The ExpandNet/WHO framework for scaling up.** Source: WHO [10]. Beginning with the end in mind: Planning pilot projects and other programmatic research for successful scaling up. WHO & ExpandNet. Geneva.

We examined the planning and management of PGB’s scaling-up process using the WHO-ExpandNet Framework. The Framework strives to improve the planning and management of the process of scaling-up of successful pilot programmes with a focus on sexual and reproductive health, making it particularly suited for an analysis of the scale-up of PGB. The Framework provides a series of recommendations for programme planning and management in order to successfully scale-up programmes [10]. According to the Framework, success of the scaling-up strategy is determined by multiple interacting factors (Figure 1). Effectiveness of a scaling-up strategy depends on the characteristics of the innovation to be scaled up, as well as the characteristics of the resource team and the user organization, each of which is influenced by the environment in which they operate. Successful management of the scaling-up process requires attention to four strategic choice areas - dissemination and advocacy, organizational processes, resource mobilization, and monitoring and evaluation. We examine each of these areas in turn with reference to PGB.

## Results

### History of PGB

Mozambique’s delegation returned from the 1994 International Conference on Population and Development (ICPD) intent on improving the situation of adolescents in their country. The need was great. Having recently emerged from a civil war that had destroyed much of the country’s infrastructure including public health capacities, Mozambique had few existing programmes specifically addressing adolescents’ sexual and reproductive health needs. In fact our search of the literature identified only one programme implemented prior to PGB that specifically addressed ASRH - Population Services International (PSI) implemented a mass media campaign in Mozambique from 1997 to 1998. In addition to social marketing of condoms and community-based activities, this intervention included HIV/AIDS behaviour-change radio spots targeted specifically at adolescents aged 13 to 20 [11]. The evaluation of this initiative found that this radio campaign reached about half of the young people it targeted but that exposure to the campaign was not associated with behaviour change [11]. A couple of additional small-scale projects that addressed elements of ASRH were implemented in Mozambique but not until after the PGB planning stages [12–15]. A more comprehensive multi-sectoral intervention including activities such as those proposed by PGB was therefore needed.

**Figure 2:**
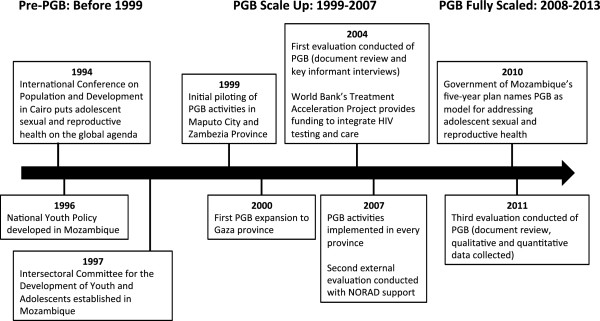
**Timeline of key events in the PGB planning, implementation, and scale-up.**

In 1997 following the ICPD, the Intersectoral Committee for the Development of Youth and Adolescents was established involving the ministries of health, education, youth, women’s affairs, labour, and environmental action as well as NGOs and faith-based organizations [1] (Figure 2). The Intersectoral Committee then led the development of the National Plan for the Development of Adolescents and Youth. Programa Geração Biz (PGB) evolved out of these efforts as a way to address the health needs of adolescents using a collaborative strategy that involved the Ministry of Health, the Ministry of Youth and Sports, and the Ministry of Education. The stated objective of PGB was “to reduce the vulnerability of adolescents and youth to poor Sexual and Reproductive Health (SRH) outcomes through the promotion of information on SRH (including HIV prevention), the adoption of skills to enable a safe transition to adulthood, and the delivery of quality clinical services, within a human rights and gender framework” [16]. These three ministries would work together with the support of UNFPA and Pathfinder International to plan and implement activities in health facilities, schools, and community settings to improve ASRH. This package of programme activities was first piloted in Maputo City and Zambezia Province starting in 1999. It was then gradually scaled-up in two ways: first, by implementing activities at additional sites within each province, and second, through expansion of the programme to new provinces. In 2000 PGB expanded to Gaza province and every year or two, additional provinces were added such that by 2007 PGB activities were being implemented in every province [5]. The number of adolescents reached by PGB increased with the expansion to new provinces: in 2000 there were 23,489 visits by young people to health facilities with PGB programmes and in 2006 this number had increased to 164,824 visits [4].

### The innovation

#### Overview of PGB

PGB adopted a three-pronged approach to reaching young people with sexual and reproductive health interventions in health clinics, schools, and the community. The clinical aspect of the programme involved integrating ASRH services into existing public-sector health facilities. Aspects of adolescent-friendly health service provision included competent and empathic staff, expanded and/or dedicated clinic hours for adolescents, offering services in a dedicated room/space within the health facility, provision of appealing health promotion information including through peer educators in waiting areas, and reduction of cost barriers [1]. The school-based aspect of the programme was implemented by peer educators (or *activistas* as they were called under the programme) and selected teachers who were trained to provide SRH education in secondary schools. Peer educators underwent 80 hours of training that prepared them to discuss early pregnancy, sexually transmitted infections (STIs) and other SRH topics with fellow students [5]. In addition to providing health education, peer educators also provided referrals to nearby adolescent friendly health clinics where students could access services [1]. Finally, out-of-school youth were trained as community-based peer educators who were responsible for reaching their out-of-school peers and others not reached through school-based programmatic activities. These community-based peer educators facilitated conversations about SRH and provided referrals to adolescent friendly health services (AFHS) [1].

#### Clarity

The intervention package was clearly defined with distinct activities and implementers. Clinical activities, including the establishment of AFHS in existing clinics, were implemented by the Ministry of Health. School-based activities were implemented by the Ministry of Education and consisted of intra and extra-curricula peer education. The Ministry of Youth and Sports spearheaded community-based activities to reach out-of-school youth. Robust referral linkages were established between these three types of activities ensuring referrals from school-based and community-based interventions to local facility-based AFHS.

#### Relevant

From its inception PGB was well placed for successful scale-up because programmatic activities composed an innovation that was *relevant* to its target population, a key characteristic identified in the WHO-ExpandNet framework [10]. A national assessment conducted by the Intersectoral Committee for the Development of Youth and Adolescents prior to the inception of PGB [1] as well as studies done at the time demonstrated the SRH needs of adolescents in Mozambique. Adolescents and those who had been pregnant as adolescents were found to be over-represented among women seeking care for complications of illegal abortion [17, 18]. Furthermore, studies conducted during this period describe high levels of pregnancy complications among adolescents including but not limited to low birth weight [19], malaria-related maternal mortality [20, 21], and maternal mortality due to eclampsia [20]. Finally, Granja and colleagues (2001) reported an adolescent maternal mortality ratio of 387 per 100,000 births compared to 294 per 100,000 births among non-adolescents [20]. These studies were sufficient to provide evidence that adolescents in Mozambique were in need of SRH interventions based on their levels of sexual activity and sexual risk behaviours, as well as their differential reproductive health outcomes compared to adult women. From its inception, PGB directly addressed many of these identified SRH needs through school, clinic, and community-based programme activities [1].

#### Credible

Furthermore, the credibility of PGB was raised through both international support and local ownership. Following the ICPD in 1994 the Intersectoral Committee for the Development of Youth and Adolescents was established in Mozambique, which led to the development of the National Plan for the Development of Adolescents and Youth, out of which came PGB [5]. PGB, therefore, came from the global agenda to advance SRH rights as established by the ICPD, and subsequently implemented by ministries of the government of Mozambique [1].

#### Compatible with local norms

Certain aspects of the Programme, such as open discussion of sexuality and SRH, particularly among unmarried youth, were not compatible with local norms. Qualitative research conducted with adolescent girls and parents in Mozambique found that there was frustration among both young people and parents about the quality of parent–child communication around sexuality and sexual risk [15]. Despite this incompatibility, the urgency of the HIV epidemic, as perceived by both the international community and within Mozambique, led to the prioritization of a risk-reduction programme despite these sensitivities.

#### Easy to install

The complexity of the multisectoral package led to some difficulties in implementing some components. Attempts were made to understand and correct implementation challenges as they arose. For example, during the course of implementation and scale-up, Pathfinder International identified challenges in recruiting and retaining female peer educators for the school-based and community-based components of PGB. In order to address this issue, the organization revised the protocol for peer-educator training and assessed the effect of the new protocol on improving retention of female peer educators [3].

#### Lessons learned

Our analysis of the characteristics of the innovation using the WHO-ExpandNet framework highlights a number of facilitators and barriers to PGB success. Contextually, PGB addressed salient ASRH needs at a time when there was political will to act on this issue due to momentum from the ICPD as well as mounting concern about the HIV epidemic. The clarity of PGB activities as well as assignment of responsibilities to the appropriate ministries facilitated implementation and encouraged local ownership within the ministries. On the other hand, the multisectoral design of the intervention introduced difficulties in coordinating and implementing the various components of PGB.

### Resource team

The *resource organizations* for this initiative were both within the Government of Mozambique and external. The Ministries of Health, Education, and Youth and Sports, the main implementers of PGB, assigned personnel who provided technical support within their respective bodies during programme implementation. The Ministry of Health oversaw clinic-based activities, the Ministry of Education was responsible for curricular and peer education initiatives in schools, while community-based activities targeting out-of-school youth fell within the purview of the Ministry of Youth and Sports [4]. This segmentation of responsibilities ensured that activities were designed and implemented by the government agency responsible for each respective sector. In addition, two international organizations, UNFPA and Pathfinder International, provided support to these three government agencies. The involvement of these agencies brought technical credibility to the initiative. Pathfinder International provided technical support to each of the three sectors of the Programme, and advisors from both organizations served on a central management committee of the Programme [4]. UNFPA provided ongoing support for Programme management and implementation, procurement, and monitoring and evaluation [9]. The two organizations were strongly committed to the Programme as further evidenced by the key role that they played in advocating for increased support for the programme from international donors including Danish International Development Agency (DANIDA), Norwegian Agency for Development Cooperation (NORAD) and Swedish International Development Agency (SIDA).

The involvement of government and international agencies in the design and management of PGB resulted in a strong set of resource organizations with the authority and the required expertise to implement an effective Programme. The government ministries brought a sound understanding of Mozambican society as well as expertise in managing their respective sectors. Pathfinder International and UNFPA were able to add their respective technical expertise in ASRH and build on experiences planning and implementing similar programmes in different contexts.

### User organization

User organizations for the Programme acted at the national, provincial, district and local levels of implementation. At the national level, the three ministries previously described as resource organizations also acted as the user organizations. The Ministry of Health organized facility-based AFHS, the Ministry of Education was responsible for the school-based component of the programme, and the Ministry of Youth and Sports led community-based initiatives [4]. Provincial management of each of the three sectors of Programme activities was conducted respectively by the Provincial Directorate of Youth and Sports, the Provincial Directorate of Education, and the Provincial Directorate of Health [4]. At the district and community levels, direct implementation of facility-based services was the responsibility of the District Directorate of Health/Community Directorate of Health while school-based activities were implemented by the District Directorate of Education/City Directorate of Education. Additional local youth associations were involved in implementation of both school-based and community-based activities [4].

#### Credibility

At the national level the three government ministries had the credibility and authority to lead this initiative. Over the course of programme implementation, PGB activities were integrated with other existing government initiatives, lending further credibility to these efforts. For example, the Ministry of Health trained a significant cadre of nurses to provide AFHS as a part of the School and Adolescent Health Programme. These providers were eventually integrated into PGB. This training conducted through the School and Adolescent Health Programme introduced the idea of AFHS to local communities lending credibility to PGB [5].

#### Capacity

Strong mechanisms were put into place to ensure adequate capacity building to establish the necessary expertise at each level of implementation. The three implementing Ministries had prior experience in programming in each of their three respective sectors. In order to strengthen their capacity for adolescent-focused SRH programme planning and implementation, they received technical support from Pathfinder International [4]. The technical advisors situated in each Ministry at the national level in turn supervised provincial technical advisers. Quarterly technical meetings at both the provincial and district levels ensured information sharing and knowledge and skills transfer among managers and direct implementers. At the district level these meetings included service providers as well as peer-educators, who were adolescents themselves [4]. As the Programme developed and scaled-up, the role of the external technical advisors was gradually scaled back, starting at the national level and progressing to the provincial level as local ownership and capacity grew to meet the needs of the Programme [5]. One of the early evaluations of PGB noted that the intersectoral approach was working well, particularly at the national and provincial levels. The evaluators noted that decision making and responsibilities for implementation were shared, such as strategic selection of sites for scaling up and delegation of sector-specific activities to the relevant ministries [7]. However, the evaluators also noted that this approach was more problematic at the district level due to sparse staffing and inadequate resources; for example, in some districts Programme activities were implemented in only two of the three sectors, reducing the overall fidelity to the planned activities [7].

#### Commitment

While UNFPA and Pathfinder International drove the planning and implementation of PGB, the Government of Mozambique showed growing commitment to the Programme at the national level. Published in 2010, the government’s five-year plan indicated that PGB was the model for addressing ASRH needs and preventing new HIV infections in adolescents [22]. The Ministry of Education, for example, provided financial support for PGB and has incorporated SRH health content into the national curriculum [5]. In the Ministry of Youth and Sports as well as the Ministry of Health, components of PGB were integrated with and supported by other policies and programmes supported by these ministries [5]. In addition, activities relating to PGB were incorporated into national, provincial and district work plans and budgets. Attempts to establish local commitment to the initiative included community sensitization involving parents, community members, and local leaders [4]. An early evaluation notes that PGB activities such as efforts to include parents in education sessions could benefit from increased coordination and systematization [7]. While in some areas Programme activities met with some initial resistance, the rapid scale-up was achieved in part through the great demand for services in areas where it had not yet been implemented [5]. In general, urban communities were more accepting of Programme activities than rural communities [5].

#### Lessons learned

One of the strengths of the multisectoral design of PGB was that each of the three ministries was responsible for Programme activities for which it was advantageously positioned to implement. Technical assistance from resource organizations provided capacity building support to the ministries in places where they were not yet equipped to implement new PGB activities. These strategies led to institutionalization of various aspects of PGB within each ministry both in terms of financial commitments from the Mozambican government and through policy action.

### Environment

The rapid scale-up of PGB was enabled by the prevailing conditions and institutions external to the programme in both national and sub-national contexts.

#### National

Several policies at the national level fostered a supportive environment for the inception and scale-up of PGB. The ICPD set the global agenda for addressing ASRH and led to the development of several supportive national initiatives including the Government of Mozambique’s National Youth Policy in 1996, and the establishment of the Intersectoral Committee for the Development of Youth and Adolescents in 1997 leading to the development of an Integrated Programme and Plan of Action to Support the Development of Adolescents and Youth [1]. Out of this plan came a needs assessment of SRH services for adolescents in Mozambique, leading to discussions on the need for intersectoral collaboration to address young people’s SRH [4]. Furthermore, programmes specifically addressing HIV and AIDS, including those set forth in the National Strategic Plan on HIV/AIDS, blazed the way in so far as they initiated dialogue among policy makers and implementers about SRH programming [1].

#### Sub-national

PGB activities were to be implemented through cooperation with local leaders and youth associations. AMODEFA, Mozambique’s International Planned Parenthood Federation member association, was to implement school and community-based activities in Maputo with collaboration with local youth associations. These youth associations were well established in Maputo prior to PGB and were well positioned to take up this role [5]. Outside of Maputo, however, youth associations were new and not well established at the time of Programme expansion, so strengthening of these groups became part of the Programme’s agenda [5]. Initial steps in scale-up included sensitization activities at the district and provincial levels to garner support for school, community, and facility-based services from local leaders and community members in order to facilitate Programme implementation [4].

#### Lessons learned

The success of PGB was partially due to the momentum from the ICPD and the ensuing national commitment to ASRH. Further success in scaling up was ensured by engaging local institutions and identifying and addressing local contextual factors to enable Programme implementation.

### Making strategic choices in scaling-up

From initial planning stages through Programme implementation, strategies for vertical and horizontal scaling up ensured sustainable installation of PGB throughout Mozambique.

#### Horizontal scaling-up

PGB was initially piloted in Maputo and Zambezia followed by staged horizontal expansion starting in 2000 when the Programme was expanded within these districts to additional provinces [1]. By 2007 PGB had initiated activities in every province in Mozambique and by 2010 activities were implemented in 83% of districts [5]. The horizontal scaling-up process was facilitated by a series of deliberate steps to ensure success and most efficient use of resources. Needs assessments were conducted to identify target provinces for expansion followed by inter-sectoral consultation to identify appropriate scopes of work within the identified provinces [4]. Throughout this process community sensitization activities were conducted with local leaders, community members, and other key stakeholders in order to garner community support and pave the way for smooth Programme roll-out [4]. Support mechanisms in each of the three sectors at the sub-national level were then established and linked vertically with national-level support as well as with other provinces with established PGB activities [4].

#### Vertical scaling-up

Components of PGB have been integrated into national policies, work plans and budgets showing strong vertical integration. With regard to clinical services, the Ministry of Health took advantage of the momentum provided by PGB activities relating to AFHS to improve pre-existing activities within the School and Adolescent Health Programme including through the training of 2,451 nurses in the provision of youth friendly health services [5]. The Ministry of Education now includes PGB content in the national curriculum, trains teachers to support peer educators, and has integrated PGB activities into its budget [5]. The Ministry of Youth and Sports has also made efforts to integrate PGB strategies into recent policies affecting adolescent health and in some provinces supports PGB coordinators [5].

### Managing the scaling up strategy

#### Dissemination and advocacy

Advocacy for PGB was conducted by Pathfinder International and UNFPA in collaboration with the Ministry of Health, Ministry of Education, and Ministry of Youth and Sports. UNFPA and Pathfinder International provided technical assistance for advocacy in order to support the institutionalization of policies supportive of ASRH [8]. Additionally these two organizations provided support to a youth advocacy group consisting of PGB peer educators from youth associations [8]. Furthermore, these agencies collaborated on a study tour for donors to see the results of PGB, resulting in support from NORAD and SIDA [4]. Certain aspects of the Programme were presented in various forums outside of Mozambique for the purpose of sharing best practices [23, 24].

#### Deciding on the managerial and organizational processes of scaling-up

**Figure 3:**
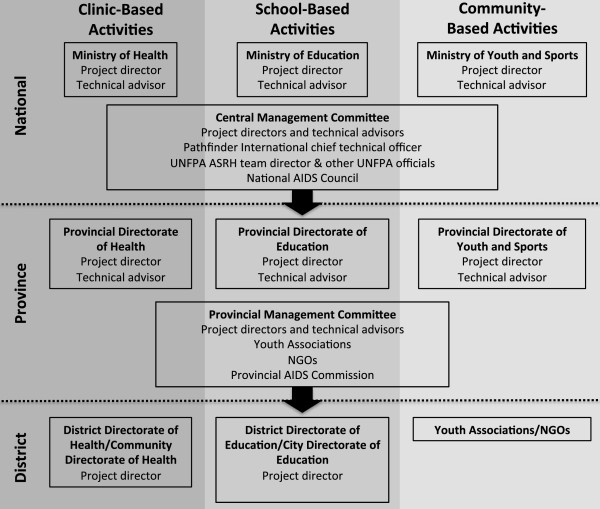
**Organizational structure of PGB management at national, province, and district levels.**

The scaling-up process for PGB relied on substantial efforts at all levels of implementation. At the national level, programme managers worked with donors to identify targets for expansion where existing physical and human resources, such as health clinics and active youth organizations, could be most efficiently harnessed [5]. Expansion to new provinces required the establishment of new provincial-level management structures, which received support vertically from the national-level management as well as horizontally from other established provincial programmes (Figure 3) [4]. At the national level, the central management committee for PGB was responsible for bringing together representatives from the three involved Ministries in order to share information and coordinate Programme planning. This structure was mirrored at the provincial level, where information was shared vertically with national-level structures as well as horizontally amongst sectors within the provincial management committee [4]. Community sensitization activities were conducted at both the province and district levels to garner support for Programme implementation [4]. Coordinators in the three sectors at the district and provincial level worked together to ensure smooth roll out of Programme activities [4]. During the course of scale-up, increased demands on provincial-level staff for activities including supervision of youth associations and peer educators, required increased staffing [5]. Gradual integration of costs and staff into the respective government sectors of Programme activities was coupled with staged reduction in technical assistance from Pathfinder International at both provincial and national levels [5]. This strategy was not without difficulties given the diversity of the settings to which PGB was scaled. In some provinces and districts the scale-up process was relatively smooth, while other struggled with issues largely related to human resources. Some provinces noted difficulties related to strained communications between sectors as well as difficulties in filling vacant technical assistance positions [7]. However, on the whole this scaled-up strategy increased the sustainability of the initiative by reducing the role of support coming from outside the government despite individual difficulties in some provinces.

#### Costs

Overall, the PGB budget appears to have been substantial. The mid-project evaluation found that the cost per young person reached by PGB had stabilized to $4.40 in 2006 [8]. Total project expenditures from 2002 through 2006 were $18.9 million while expenditures in 2005 through 2009 were $27.2 million, an increase in line with the substantial scale-up that had occurred during the decade [9]. Funding for PGB activities came from a variety of international funding bodies as well as from the government of Mozambique. UNFPA and Pathfinder International took the lead in organizing donor support for the scale up [4]. Primary external donors included DANIDA, NORAD, SIDA, and Trocaire [5]. External funds were disbursed by UNFPA to the provinces for implementation of Programme activities [4]. Some funding came from within the Government of Mozambique, from a number of Ministries [4, 6]. Other funders have provided support to pieces of the initiative specific to the interest areas of the funding organizations. For example, the World Bank’s Treatment Acceleration Project provided $1.25 million over four years starting in 2004 to integrate HIV testing and care into AFHS in Maputo and Gaza Province. Through this Project more than 600 new Adolescent HIV cases were identified and adherence support efforts resulted in high retention in antiretroviral therapy [25]. USAID’s Interagency Gender Working Group, UNFPA, and the Flanders Government provided support for improving recruitment and retention of female peer educators [4, 26]. The Flanders Government also funded an initiative to integrate a response to gender-based violence into existing PGB structures [4].

#### Monitoring and evaluation

A strong emphasis was placed on monitoring and evaluation (M&E) throughout the planning and implementation of PGB. The initial Programme objectives included a mandate to build the capacity of user organizations to monitor and evaluate implementation of the Programme [1]. Monitoring of Programme activities occurred in each of the sectors of implementation. For example, within the education sector, peer-educator recruitment, training, and activities were tracked [1]. One of the early external evaluations indicated that while this was generally an acceptable system, pieces of it were perceived by some stakeholders as overly complicated and some others had not received necessary training for completing monitoring forms [7]. Linkages between the sectors of implementation were also monitored by recording referrals from community-based activities to facility-based services [1]. Throughout Programme implementation the monitoring and evaluation system evolved. A large amount of data was lost in 2006, after which the Management Information System was redesigned [8]. The later 2007 external evaluation indicated that the M&E system was performing well in collecting Programme implementation data, but could improve in documenting capacity building, advocacy, and similar activities [8]. Furthermore, this evaluation noted that M&E efforts had focused on clinic-based service delivery, while other aspects, including peer education, advocacy, and capacity building, were not as closely monitored [8]. The final 2011 external evaluation noted weakness within the M&E systems in many of the provinces, largely due to rapid turnover of project staff, which contributed to late or absent Programme reports [9].

In addition to these internal mechanisms for M&E, three external evaluations were carried out over the course of the scale-up of PGB to assess the Programme’s implementation, reach, and effect on multiple sexual and reproductive health outcomes. An evaluation in 2004 included a review of Programme documentation as well as interviews with individuals at all levels of Programme implementation [7]. In 2007 an external evaluation was conducted with the support of NORAD. This evaluation consisted of key informant interviews and a review of Programme documentation [8]. A third evaluation was conducted in 2011, which involved document review as well as both qualitative and quantitative data collection from Programme beneficiaries [9].

#### Lessons learned

Successful scaling-up requires adequate resources to support making strategic decisions for where to expand, conduct community mobilization, and train and support new Programme staff at multiple levels within each ministry. While emphasis was placed on M&E, the Programme could have benefitted from an evaluation strategy from the outset that examined changes in health behaviours and clinical outcomes as the Programme expanded to new areas.

### PGB impact on behaviors and outcomes

Evaluating the impact of PGB is a challenging task for several reasons. First, the Programme reached national coverage raising difficulties in identifying appropriate comparison groups for evaluation. Second, it addressed numerous social and health outcomes, targeted multiple distinct populations, and was implemented in a variety of settings. In order to assess PGB’s impact we provide a review of the results of research studies on ASRH in Mozambique that were conducted during and after the scale-up of PGB. Most of these studies were not specifically conducted as evaluations of PGB so we cannot directly or entirely attribute their results to PGB activities. However, this approach provides an overview of the changing landscape of the state of ASRH in the country as PGB was scaled up to the national level.

#### Adolescent sexual and reproductive health during PGB piloting and scale-up (1999–2007)

During the years of PGB scale-up poor SRH outcomes, such as high rates of early pregnancy and incident STIs, and low use of SRH services remained salient. Analyses of the country’s 2003 Demographic and Health Survey data indicated that 10% of girls gave birth by the age of 15 years [27]. Secondary analysis of surveillance data showed that from 2000 to 2004 the HIV prevalence in Mozambique among young women ages 15 to 24 was gradually increasing [28]. Nationally representative data collected in 2001 indicated that among sexually experienced youth aged 15 to 24 about 20% had a history of STI symptoms while less than 4% had ever used HIV counseling or testing services [29]. In qualitative research conducted in 2002, adolescents in Maputo indicated a scarcity of information on SRH provided in school and expressed a desire for more comprehensive instruction on these matters [30].

Furthermore, additional research highlighted the ongoing behavioral trends in sexual activity and condom use that are intermediate to SRH outcomes. Secondary analysis of Demographic and Health Survey data revealed that early sexual debut increased from 1997 to 2003 among adolescent boys [28]. A 2007 survey of in-school youth found that 27% of male students and 22% of female students aged 16 had ever experience forced or coerced sex [31]. And condom use at last sex among sexually active young people ranged from 20-30% in surveys conducted in 2001 and 2003 [28, 32].

In addition to the general research on ASRH, several studies specifically acknowledged PGB activities, though none provide extensive evaluation. Melo and colleagues surveyed young women attending a PGB clinic in 2002 with encouraging results [33]. About three quarters of those who had casual sexual partners reported condom use at last sex [33]. STI rates were similar to or lower than those reported in other studies conducted in Mozambique [33]. Furthermore, these young women had relatively high-levels of correct knowledge about STI prevention and somewhat lower levels of STIs when compared to other studies conducted in Mozambique [33]. While this study provides several interesting insights into the users of the clinics supported by PGB, it is not possible to determine if the positive indicators, such as high condom use and low STI prevalence, were due to intervention activities or due to self-selection of service users. Groes-Green conducted ethnographic fieldwork among youth in Maputo and published several papers that comment on the peer education and school-based components of PGB [34, 35]. He observed gender equitable discussions of sex and sexuality among male and female students and teachers [35].

Additional insights into possible PGB impact can be gleaned from quantitative surveys that were conducted in 2003 and 2005 in several provinces as part of PGB evaluation activities and reported in the grey literature [4]. Notably, the proportion of respondents who used contraception at first sexual intercourse increased from 36% in 2003 to 60% in 2005. Condom use within committed relationships increased from 70% to 83% and the percent who had taken an HIV test increased from 11% to 38% from 2003 to 2005 [4].

#### Adolescent sexual and reproductive health after PGB scale-up (2008–2013)

A Programme evaluation conducted in 2011 included an additional quantitative survey of PGB’s target population and examined knowledge, attitudes, and behaviours by exposure to PGB activities [9]. The proportion of respondents who used contraception was somewhat higher among those who had been exposed to PGB (57%) compared to the overall study population (53%); knowledge of modern contraceptive methods was higher among those exposed to the PGB as well, possibly indicating a positive intervention effect. PGB exposure also appeared to increase use of healthcare services, as adolescent girls in the survey were more likely to access AFHS if they had been exposed to PGB activities [9]. Respondents exposed to PGB also had somewhat more positive attitudes toward condom use. For example, they were more likely to agree that using condoms with a new sexual partner is a good practice [9].

Data from Demographic and Health Surveys show some indications of overall reductions in adolescent fertility. From 1997 to 2003 fertility among urban adolescents aged 15–19 decreased from 175 to 143 births per thousand adolescents [36, 37]. The fertility rate in rural areas decreased somewhat later, possibly corresponding to later scale-up of PGB activities. In 2003 the rural fertility rate among adolescents aged 15–19 was 207 while in 2011 it had dropped to 183 births per thousand adolescents [36, 38].

Independent research conducted in Mozambique after the completion of PGB scale-up indicates that there is much work to be done. Condom use among sexually active adolescents ranges from low to moderate [39]. Modeling estimates indicate no statistically significant change in the HIV prevalence among women ages 15–24 at antenatal care [40]. A study conducted in Beira from 2009 to 2012 found that among older high-risk adolescent girls (those with multiple partners in the previous month) HIV prevalence was almost 20% [41]. Clearly the programmatic activities of PGB remain relevant and on-going efforts are needed to sustain and adapt interventions to the evolving environments and needs of adolescents.

## Discussion

In our review of Programme reports and evaluations we found that many elements of PGB were planned and implemented in ways that optimally situated it for successful scaling up. The momentum from the ICPD translated to a priority within the Mozambican Government to form policies that promoted ASRH. The design of the Programme and the package of PGB activities came out of this agenda and formed an innovation that was clear and credible, though there were difficulties in relation to compatibility with local norms and ease of installation, due to the complex multisectoral nature of PGB. Successful management and scale-up can be credited to a cohesive resource team that supported the national and sub-national agencies that served as the user organizations. UNFPA and Pathfinder International built capacity within the Ministries of Health, Education, and Youth and Sports so that they could implement Programme activities within their respective sectors. The three Ministries demonstrated some commitment through integrated policies and budgetary allocations. The vertical and horizontal scaling up of PGB was undertaken deliberately through coordinated action on multiple levels of governance and within each of the three participating Ministries. Furthermore, efforts were made throughout the scale-up to document progress through M&E.

The multisectoral approach was entirely appropriate to address ASRH in the Mozambican context and may translate well to other settings. Adolescents were reached in clinics, schools, and communities—settings that are important for young people globally. In Mozambique, the three Ministries that had expertise in providing services in each of these three settings were identified and their unique capacities were leveraged and enhanced. While the multisectoral strategy can draw on these strengths, it also requires commitment of adequate resources to build capacity and provide linkages and coordination between sectors at every level of implementation. This support can be provided by international NGOs and UN agencies but efforts should be made to build government capacity and gradually transition to independent implementation.

The WHO-ExpandNet framework provides a valuable tool for identifying elements of programmes that contribute to or impede successful scaling up. In the case of PGB we found that the management structures and systems that were put in place contributed to successful implementation of a multicomponent intervention by taking advantage of the unique strengths of three different Ministries, which acted as user organizations. Furthermore, gradual horizontal and vertical scaling up was successful due to deliberate efforts to build capacity and institutionalize aspects of the Programme. Furthermore, close attention was paid to monitoring and evaluation so that operations could be modified as challenges arose in the process of scaling up. External evaluations, which were conducted periodically, served as feedback mechanisms and led to improvements within these internal M&E systems.

While PGB appears to have successfully provided SRH information and services to many adolescents in Mozambique, it did not sufficiently address social norms that likely contribute to ASRH outcomes. Concerns relating to gender equity were raised in routine monitoring when it was found that adolescent boys outnumbered girls among PGB peer educators [26]. While modifications were made specifically to the peer-education training protocol to address this issue [26], more could have been done, especially with families and communities, to address social norms about gender and SRH. Inadequate gender sensitivity may have contributed to the limited effect PGB had on preventing unprotected sexual activity in girls.

There are some limitations to this analysis that should be noted. Evaluation reports of PGB exist largely in the grey literature. Much of our assessment of Programme implementation and outcomes relies on these reports as well as ancillary studies that were not intended to directly evaluate PGB. Despite this limitation, we feel that there is ample evidence to facilitate a discussion of the processes of PGB planning, implementation, and scale up. This paper adds to the evidence for successful programmatic elements of planning for scale-up of ASRH programmes, which includes documentation of the scaling up of comprehensive sexuality education in Nigeria [42]. Furthermore, lessons from the successes and challenges can be used in the planning of future large-scale public health interventions.

## Endnote

^a^In Portugese, *geração biz* is slang for “busy generation.”
